# The effect of vitamin C on sociability in a juvenile zebrafish pesticide-induced model of autism spectrum disorder

**DOI:** 10.1192/j.eurpsy.2021.548

**Published:** 2021-08-13

**Authors:** M.A. Robea, A. Ciobica, S.-A. Strungaru, G. Plavan, M. Nicoara, C. Solcan

**Affiliations:** 1 Department Of Biology, “Alexandru Ioan Cuza” University of Iasi, Faculty of Biology, Iasi, Romania; 2 Department Of Research, “Alexandru Ioan Cuza” University of Iasi, Faculty of Biology, Iasi, Romania; 3 Center Of Biomedical Research, Romanian Academy, Iasi, Romania; 4 Department Of Interdisciplinary Research In Science, “Alexandru Ioan Cuza” University of Iasi, Iasi, Romania; 5 Department Of Molecular Biology, Histology And Embriology, University of Agricultural Science and Veterinary Medicine “Ion Ionescu de la Brad”, Faculty of Veterinary Medicine, Iasi, Romania

**Keywords:** vitamin C, pesticide, autism spectrum disorder, sociability

## Abstract

**Introduction:**

Autism spectrum disorder (ASD) is a multi-factorial disease characterized by impairments in social interaction, communication and repetitive behaviors. The necessity of developing an adequate treatment for ASD is essential. There is an increase in clinical studies assessing the positive effects of vitamins in ASD children. Vitamin C (vit. C) is implicated in biosynthesis of neurotransmitters and in protein metabolism.

**Objectives:**

This study evaluated the possible effect of vit. C on zebrafish sociability after a single insecticide mixture administration as inductor for ASD.

**Methods:**

A single dose of insecticide mixture (600 μg L^-1^ fipronil and 600 μg L^-1^ pyriproxyfen) was administrated to zebrafish juvenile. Vit. C (25 μg L^−1^) was daily administrated during 14 days. A control group simulated the administration of insecticide mixture and vitamin. Each animal was tested in the experimental tank designed for the social interaction test. The trials were recorded and analysed using EthoVision XT 11 (NOLDUS, Netherlands). The locomotor activity parameters and the time spent next to the group were measured. Each trial had 4 minutes duration.

**Results:**

We have found no significant differences in the average levels between pre-treatment and treatment days (P< 0.05 ANOVA) regarding the locomotor activity parameters. Significant changes in sociability were observed for the group exposed to insecticide mixture and for vit. C group (P > 0.05 ANOVA). It was also found that 14 days vitamin administration can lead to sociability improvements after a single administration of mixture insecticide.

**Conclusions:**

The results of the current study bring some positive insights for the future of ASD therapy.

**Conflict of interest:**

This work was co-funded by the European Social Fund, through Operational Programme Human Capital 2014-2020, project number POCU/380/6/13/123623, project title

